# Supplementary material for: Inflammatory factors and risk of lung adenocarcinoma: a Mendelian randomization study mediated by blood metabolites
Source: Front Endocrinol (Lausanne). 2024 Aug 27;15:1446863. doi: 10.3389/fendo.2024.1446863 (PMC11384989; doi:10.3389/fendo.2024.1446863)
Supplement: Supplementary file 1 [file DataSheet1.zip › supplementary files/S2/GCST90200696/forest.pdf]

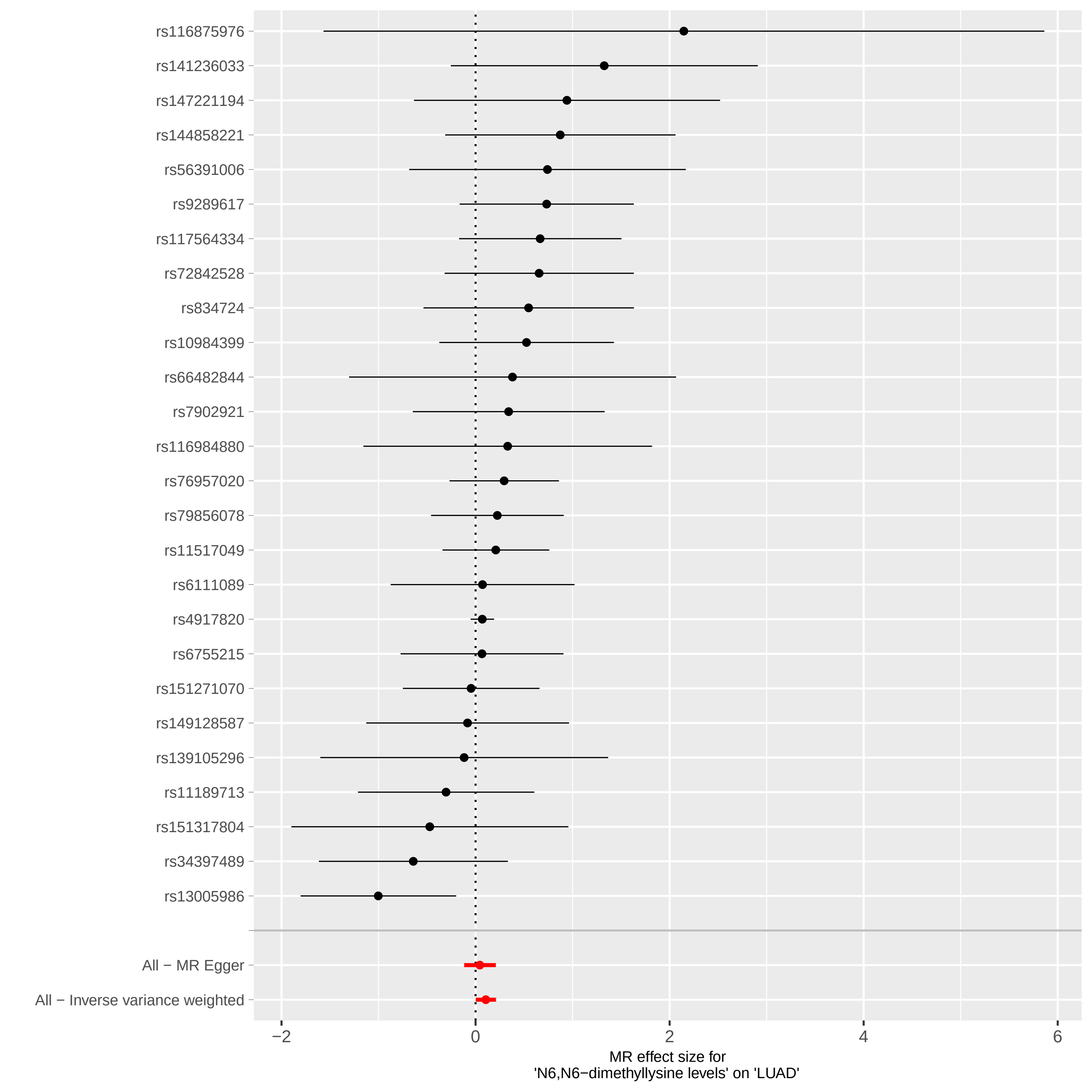
rs116875976
rs141236033
rs147221194
rs144858221
rs56391006
rs9289617
rs117564334
rs72842528
rs834724
rs10984399
rs66482844
rs7902921
rs116984880
rs76957020
rs79856078
rs11517049
rs6111089
rs4917820
rs6755215
rs151271070
rs149128587
rs139105296
rs11189713
rs151317804
rs34397489
rs13005986
All − MR Egger
All − Inverse variance weighted
−2
0
2
4
6
MR effect size for
'N6,N6−dimethyllysine levels' on 'LUAD'
